# Breeding progress for pathogen resistance is a second major driver for yield increase in German winter wheat at contrasting N levels

**DOI:** 10.1038/s41598-020-77200-0

**Published:** 2020-11-23

**Authors:** Holger Zetzsche, Wolfgang Friedt, Frank Ordon

**Affiliations:** 1 Julius Kuehn Institute (JKI), Federal Research Centre for Cultivated Plants, Institute for Resistance Research and Stress Tolerance, 06484 Quedlinburg, Germany; 2 iFZ Research Centre for Biosystems, Land Use and Nutrition, Department of Plant Breeding, Justus Liebig University, 35392 Giessen, Germany

**Keywords:** Plant breeding, Biotic

## Abstract

Breeding has substantially increased the genetic yield potential, but fungal pathogens are still major constraints for wheat production. Therefore, breeding success for resistance and its impact on yield were analyzed on a large panel of winter wheat cultivars, representing breeding progress in Germany during the last decades, in large scale field trials under different fungicide and nitrogen treatments. Results revealed a highly significant effect of genotype (G) and year (Y) on resistances and G × Y interactions were significant for all pathogens tested, i.e. leaf rust, strip rust, powdery mildew and Fusarium head blight. N-fertilization significantly increased the susceptibility to biotrophic and hemibiotrophic pathogens. Resistance was significantly improved over time but at different rates for the pathogens. Although the average progress of resistance against each pathogen was higher at the elevated N level in absolute terms, it was very similar at both N levels on a relative basis. Grain yield was increased significantly over time under all treatments but was considerably higher without fungicides particularly at high N-input. Our results strongly indicate that wheat breeding resulted in a substantial increase of grain yield along with a constant improvement of resistance to fungal pathogens, thereby contributing to an environment-friendly and sustainable wheat production.

## Introduction

Innovations in resistance to pathogens have been a major goal in wheat breeding as plant resistance is the most environment-friendly and cost-efficient way of plant protection. Together with higher yield potential and enhanced production systems improved resistance has resulted in an average yield increase of 1–2% per year on a global scale over the last 50 years^1^. However, cereal rusts (*P. striiformis*, *P. triticina*), powdery mildew (*Blumeria graminis*), and *Fusarium* sp. remain major fungal wheat pathogens in temperate regions including Germany^2–5^. Each of these pathogens is a threat to wheat production, causing yield losses of up to 70% in susceptible cultivars^6,7^. Nonetheless, devastating fungal epidemics were prevented in Europe including Germany and elsewhere in the last decades by the use of fungicides and resistant cultivars^8^.

*Puccinia striiformis* Westend f.sp. *tritici* (PS) is the causal agent of stripe or yellow rust (YR). With the rise of the “Warrior” and “Kranich” races, YR has again gained importance also in Germany since 2011 most likely due to changes in the adaptation of *P. striiformis* to higher temperatures^9,10^. Moreover, the pathogen is expected to further benefit from climate change accompanied by rising ambient winter temperature and its long-distance migration capacity^11,12^. Nitrogen (N) fertilization affects the level of YR infestation, with higher N increasing disease severity depending on the cultivar and environmental conditions^13–15^.

*Puccinia triticina* Erikss. (PT) causing leaf rust (LR) is the most widespread and economically important wheat rust worldwide^16–18^ although its importance decreased slightly in Germany in recent years due to the abundance of YR^19^. Races of *P. triticina* recently prevalent in Germany carry many different virulence genes against most of the resistance genes used in elite wheat cultivars^20,21^. An increasing severity of LR was observed along with the rate of N applied^22–24^.

Similar to the rusts many different isolates are known for *Blumeria graminis* (DC.) Speer f.sp. *tritici* (BGT), the causal agent of powdery mildew (PM). Various *Pm* genes have been introduced from older wheat cultivars or land-races^25,26^ or were transferred from ancestral wheat and closely related wild species such as *T. monococcum*^27^, *Aegilops speltoides*^28^, or *Ae. tauschii*^29,30^. Due to frequent sexual recombination, regional populations of BGT are highly diverse, very dynamic in their virulence pattern, and fastly adapting to the host genotype^31,32^.

*Fusarium culmorum* (W.G. Sm.) Sacc. (FC), one of the pathogens that can cause Fusarium head blight (FHB) or scab, has no race differentiation. FHB resistance can be characterized as resistance to initial penetration (type I) and resistance to spreading of the pathogen within the host tissue (type II)^33,34^. Further types of *Fusarium* resistance refer to the infection of grains, including resistance against kernel infestation, tolerance to yield loss, resistance against mycotoxin accumulation in grains, and resistance against alteration of grain components^35–37^. FHB resistance seems to be controlled by a small number of major genetic loci (major QTL) and many minor QTL being largely influenced by the environment^38,39^. Findings of the effect of N supply on FHB severity are inconsistent. Some authors reported an increase in FHB infestation with N supply^40,41^, while others found inconsistent results^42^ or no significant effect^43,44^ at different N levels.

With respect to yield a stagnation has been observed since the beginning of the twenty-first century in Western Europe including Germany^1^ as a consequence of the extension of wheat cultivation to marginal soils, restricted nitrogen (N) input and pesticide use, more volatile weather conditions, and an increase of organic farming^45^. Yield gains in most developed and developing countries increased significantly in the last 4 to 5 decades, ranging from 28 to 135 kg ha^−1^ a^−1^^46–50^. To quantify breeding progress in German winter wheat varieties released between 1966 and 2007 were investigated by Ahlemeyer and Friedt^51,52^. Yield progress was between 30.7 kg ha^−1^ a^−1^ (including plant protection) and 32.2 kg ha^−1^ a^−1^ (without plant protection) mainly resulting from a higher number of kernels per spike (KPS). Significant progress in resistances to fungal pathogens was made as indicated by decreasing susceptibility for PM and LR. Further studies of long-term breeding progress in German winter wheat yield revealed genetic gains of 51 kg ha^−1^ a^−1^^53^ and relative genetic gains of 0.66% a^−1^ at low intensity and 1.16% a^−1^ at high intensity^54,55^. Ahrends et al.^56^ found a treatment-specific linear yield increase ranging from 25 to 32 kg ha^−1^ a^−1^ of 16 cultivars released in Germany between 1895 and 2007 if treated with combined synthetic-organic fertilizer but no significant progress in non-fertilized conditions.

While it is generally agreed that wheat has been improved towards more durable resistances against fungal pathogens over the last 50 years^7,57^, studies to quantify this efforts and its impact on grain yield have not been published. Voss-Fels et al.^58^ intensively investigated breeding progress in economically successful European winter wheat with respect to production intensity. This study aims to complement the former general analysis based on the subset of economically important German cultivars. The objectives were to quantify the breeding progress with respect to resistance to major fungal pathogens and its effect on grain yield in relation to (i) nitrogen fertilization, (ii) fungicide treatment, (iii) and year of cultivar release.

## Results

### Reaction of winter wheat varieties to major fungal diseases

Stripe or yellow rust (YR) was the most prevalent disease with a three-year´s mean score of infection (mean of annual average ordinates, AO) of 7.8 ± 6.8% leaf area infected, followed by leaf rust (LR) 6.9% ± 4.9%, and powdery mildew (PM) with 4.9 ± 3.4%. Fusarium head blight (FHB) was scored with an average of 2.6 ± 1.8% ear area infected. N fertilization (T1 vs. T3) significantly increased the susceptibility to biotrophic pathogens (YR + 7.7%, LR + 23.6%, PM + 23.3%) as well as to the hemibiotrophic pathogen (FHB + 33.3%). Summary statistics, orthogonal contrasts between treatments and ANOVA results are shown in Table 1. Genetic variation of cultivars (G) to all fungal diseases was highly significant (*p* < 0.001). Nitrogen (N) and year (Y) as well had a highly significant impact on the susceptibility to all fungal pathogens. Relative importance of diseases differed among years as indicated by significant G × Y interaction (above average: 2015 YR; 2016 FHB; 2017 LR, FHB, PM). G × N and G × N × Y interactions were significant for FHB only.

**Table 1:** Summary statistics of disease scores (average ordinate, AO) for four fungal pathogens, orthogonal contrasts between treatments based on LSmeans and ANOVA results for 3-year field trials 2014/15, 2015/16, 2016/17 obtained on 178 wheat cultivars.

Disease/observations (N)	YR	LR	PM	FHB
	2136	2136	2136	2136
Treatment	∅ ± SD	∅ ± SD	∅ ± SD	∅ ± SD
T1	7.55 ± 7.16	6.19 ± 4.29	4.43 ± 3.13	2.22 ± 1.54
T2				
T3	8.13 ± 6.46	7.65 ± 5.28	5.46 ± 3.58	2.96 ± 1.94
T4				
Total	7.84 ± 6.82	6.92 ± 4.86	4.94 ± 3.40	2.59 ± 1.79

Orthogonal contrasts	p > F signif. p	p > F signif. p	p > F signif. p	p > F signif. p
T1 vs. T3 (N rate)	0.0492*	< 0.0001***	< 0.0001***	< 0.0001***

ANOVA source of variation	p > F signif. p	p > F signif. p	p > F signif. p	p > F signif. p
Genotype (G)	< 0.0001***	< 0.0001***	< 0.0001***	< 0.0001***
Nitrogen (N)	< 0.0001***	< 0.0001***	< 0.0001***	< 0.0001***
Year (Y)	< 0.0001***	< 0.0001***	< 0.0001***	< 0.0001***
G × Y	< 0.0001***	< 0.0001***	< 0.0001***	< 0.0001***
G × N	0.9758 ns	0.9996 ns	1.0000 ns	0.0091**
G × N × Y	0.8417 ns	1.0000 ns	1.0000 ns	0.0005***

YR, stripe rust, % diseased leave area (Average Ordinate, AO); LR, leaf rust, % diseased leave area (AO); PM, powdery mildew, % diseased leave area (AO); FHB, Fusarium head blight, % diseased ear area (AO); T1–T4, treatments T1 (target supply 110 kg N ha^−1^ + inoculation), T2 (target supply 110 kg N ha^−1^ + fungicides), T3 (target supply 220 kg N ha^−1^ + inoculation), T4 (target supply 220 kg N ha^−1^ + fungicides); ∅, mean; SD, standard deviation; Signif. p, significance level of p value: *** < 0.001, ** < 0.01, * < 0.05, ns not significant.

### Grain yield and yield components

Mean grain yield (GRY) across all treatments was 6.4 ± 2.0 t ha^−1^ (Table 2). Regarding yield components, mean thousand kernel weight (TKW) was 34.6 ± 5.9 g, kernels per spike (KPS) 38.7 ± 11.7, ears per square meter (ESM) 492 ± 84 ears m^−2^, biomass (BM) 17.8 ± 3.6 t ATM ha^−1^, and harvest index (HI) 0.34 ± 0.06 (Table 2). GRY, BM and all direct yield components showed substantial genetic variation (G, p ≤ 0.001). Treatments resulted in highly significant differences for GRY and most of the yield components except ESM as direct comparisons by orthogonal contrasts show (Tables 1, 2). Plant protection (PP, T1, T3 vs. T2, T4) significantly increased BM (+ 20.8%), GRY (+ 40.9%), and all yield components (TKW + 20.0%, KPS + 17.3%, ESM + 1.1%), as well as HI (+ 15.9%). Elevated N fertilization (T1, T2 vs. T3, T4) resulted in significantly higher BM (+ 4.3%) and ESM (+ 2.4%), whereas TKW (− 8.8%), HI (− 8.6%), and GRY (− 3.4%) were significantly decreased. KPS (+ 1.7%) was not significantly affected by N application. While a higher N supply together with plant protection results into higher GRY (T2 7.4 t ha^−1^ vs. T4 7.7 t ha^−1^), higher susceptibility to fungal pathogens at a high N rate resulted in lower GRY (T1 5.7 t ha^−1^ vs. T3 5.0 t ha^−1^). Increased GRY due to higher N under plant protection (T4 vs. T2) was much less than expected. While lodging (T4 > T3 > T2 >  > T1) might have contributed to lower GRY at higher N the results can be explained by the influence of the year. Year (Y) significantly affected GRY, BM, and all yield components mainly due to annual differences in the intensity of drought periods during spring and summer. While GRY (7.8 t ha^−1^), KPS (45.6), BM (19.1 t ATM ha^−1^), and HI (0.37) were highest in 2016, the year 2015 with the strongest drought resulted in the lowest average performance (GRY 5.2 t ha^−1^, BM 15.2 t ATM ha^−1^, KPS 28.7, HI 0.30). ESM was significantly increased in 2015 (538 ears m^−2^) likely resulting from high N_min_ and above average precipitation in winter 2014/15. N application was likely only partly successful in the drought stress years 2015 and 2017. Moreover, early N application might have been contributed to drought stress by higher tiller competition (ESM higher in T3 and T4 than in T1 and T2, c.f. Tab. 2). Higher N together with higher soil moisture in 2016 resulted in a more normal 14% increase in GRY in T4 vs. T2. Stressed conditions particularly at higher N rates in both drought years are also supported by harvest index (HI). While in general low in the drought stress years, HI was higher in T2 vs. T4 in 2015 (0.313 vs. 0.297) and 2017 (0.399 vs. 0.358). It was opposite in 2016 (T2: 0.378 vs. T4: 0.409). Higher N application was in general more successful with regard to crude protein (+ 10.4% in T4 vs. T2 over all three years, data not presented in the manuscript). Y also affected TKW, ESM, BM and HI of single genotypes as indicated by significant G × Y interaction, which was not the case for KPS and GRY.

**Table 2:** Summary statistics, orthogonal contrasts between treatments based on LSmeans and ANOVA results of yield and yield components and ANOVA results with significance levels of 3-year field trials (2014/15, 2015/16, 2016/17) obtained on 178 wheat cultivars.

Yield parameter observations (N)	GRY	TKW	KPS	ESM	BM	HI
	4272	4272	4264	4264	4272	4272
Treatment	∅ ± SD	∅ ± SD	∅ ± SD	∅ ± SD	∅ ± SD	∅ ± SD
T1	5.73 ± 1.18	33.5 ± 4.5	36.6 ± 8.8	483 ± 89	16.0 ± 2.3	0.335 ± 0.045
T2	7.36 ± 1.85	38.9 ± 5.3	40.2 ± 11.9	490 ± 88	18.8 ± 3.4	0.365 ± 0.058
T3	4.95 ± 1.40	29.4 ± 4.8	34.7 ± 9.2	496 ± 78	16.2 ± 3.1	0.286 ± 0.056
T4	7.69 ± 2.17	36.6 ± 4.4	43.4 ± 14.0	500 ± 79	20.1 ± 3.8	0.355 ± 0.065
Total	6.43 ± 2.04	34.6 ± 5.9	38.7 ± 11.7	492 ± 84	17.8 ± 3.6	0.335 ± 0.064

Orthogonal contrasts	p > F signif. p	p > F signif. p	p > F signif. p	p > F signif. p	p > F signif. p	p > F signif. p
T1 vs. T2 (protection at low N)	< 0.0001***	< 0.0001***	< 0.0001***	0.0703 ns	< 0.0001***	< 0.0001***
T3 vs. T4 (protection at high N)	< 0.0001***	< 0.0001***	< 0.0001***	0.2023 ns	< 0.0001***	< 0.0001***
T1 vs. T3 (N rate unprotected)	< 0.0001***	< 0.0001***	< 0.0001***	0.0007***	0.1321 ns	< 0.0001***
T2 vs. T4 (N rate protected)	< 0.0001***	< 0.0001***	< 0.0001***	0.0041**	< 0.0001***	< 0.0001***
T1, T2 vs. T3, T4 (N rate)	< 0.0001***	< 0.0001***	0.0533 ns	< 0.0001***	< 0.0001***	< 0.0001***
T1, T3 vs. T2, T4 (protection)	< 0.0001***	< 0.0001***	< 0.0001***	< 0.0292*	< 0.0001***	< 0.0001***

ANOVA source of variation	p > F signif. p	p > F signif. p	p > F signif. p	p > F signif. p	p > F signif. p	p > F signif. p
Genotype (G)	< 0.0001***	< 0.0001***	< 0.0001***	< 0.0001***	< 0.0001***	< 0.0001***
Nitrogen (N)	< 0.0001***	< 0.0001***	0.0063**	< 0.0001***	< 0.0001***	< 0.0001***
Plant protection (PP)	< 0.0001***	< 0.0001***	< 0.0001***	0.0084**	< 0.0001***	< 0.0001***
Year (Y)	< 0.0001***	< 0.0001***	< 0.0001***	0.0027**	< 0.0001***	< 0.0001***
G × Y	0.1872 ns	< 0.0001***	0.7066 ns	< 0.0001**	0.0001***	< 0.0001***
G × N	1.0000 ns	1.0000 ns	0.9988 ns	0.9652 ns	0.9942 ns	0.0018**
G × PP	< 0.0001***	0.0003***	0.2824 ns	0.0003***	< 0.0001***	0.0001***
G × N × PP	1.0000 ns	1.0000 ns	0.8122 ns	0.8722 ns	0.9902 ns	1.0000 ns
G × N × PP × Y	1.0000 ns	1.0000 ns	1.0000 ns	0.9901 ns	1.0000 ns	1.0000 ns

GRY, grain yield in t/ha (average moisture 14%); TKW, 1000 kernel weight in g; KPS, kernels per spike; ESM, stand density/m^2^ (productive spike numbers/m^2^); BM, aboveground biomass (ATM) in t/ha; HI, harvest index; T1–T4, treatments T1 (target supply 110 kg N ha^−1^ + inoculation), T2 (target supply 110 kg N ha^−1^ + fungicides), T3 (target supply 220 kg N ha^−1^ + inoculation), T4 (target supply 220 kg N ha^−1^ + fungicides); ∅, mean; SD, standard deviation; Signif. p, significance level of p value: *** < 0.001, ** < 0.01, * < 0.05, ns not significant.

Genotype × plant protection (G × PP) interaction was significant for GRY, TKW, ESM, BM, and HI but not for KPS. G × N interaction was only significant for HI. The three-way G × PP × N rate interaction and the four way-interaction (G × PP × N × Y) were not significant for any trait.

### Impact of major diseases and genotypic resistance on yield and yield components

**Figure 1:**
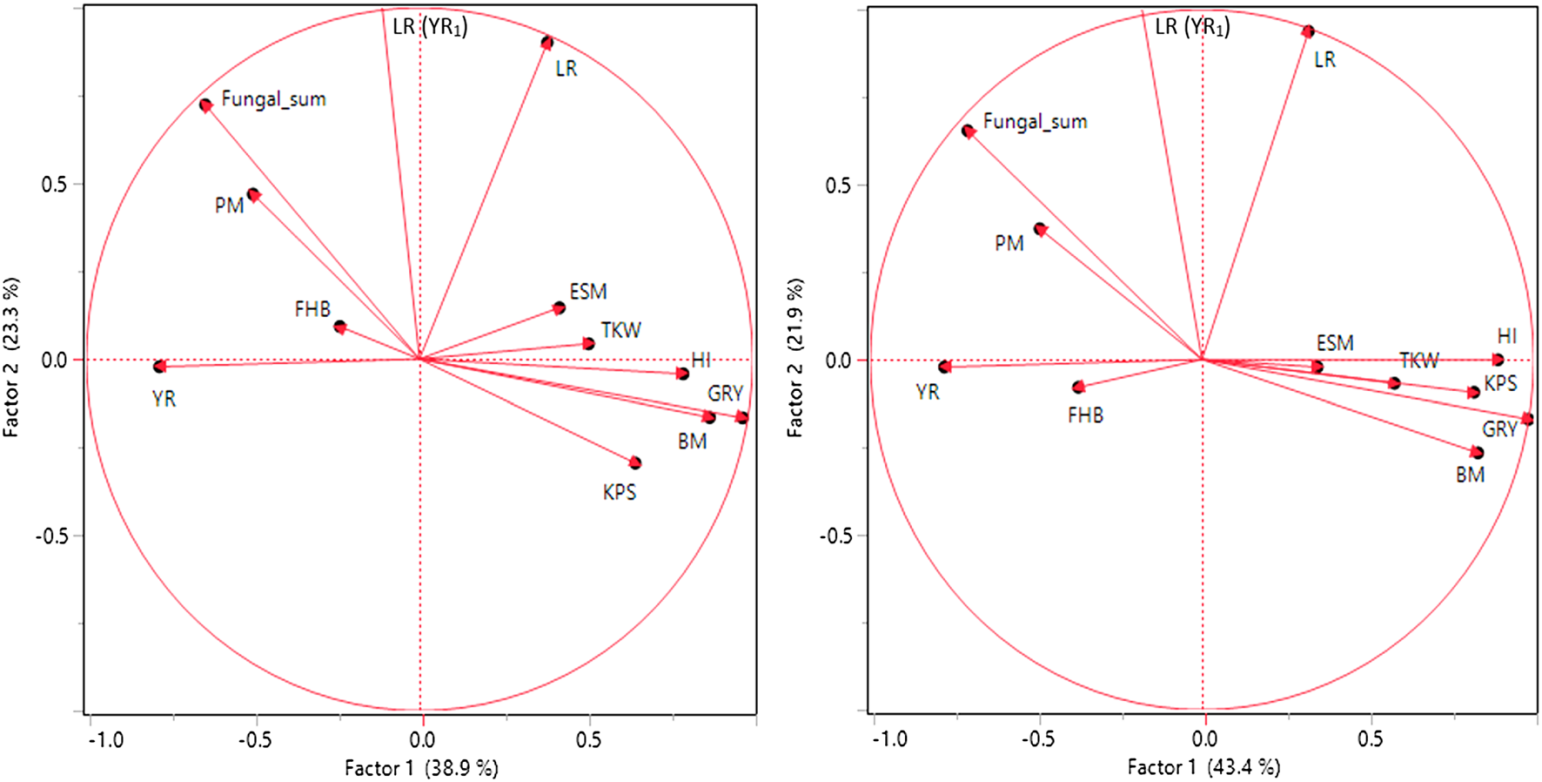
Factor analysis by means of principal component analysis (PCA) of fungal diseases (YR, LR, including subgroup LR (YR_1_) with low YR infestation < 4.05%, PM, FHB, Fungal sum), grain yield (GRY), and yield components (TKW, KPS, ESM, BM, HI) based on LSmeans (treatment-specific, over years and repeats) at two N levels (left 110 kg N ha^−1^, right 220 kg N ha^−1).^ Factors PC1 and PC2 explained 38.9% (43.4%) and 23.3% (21.9%) of the phenotypic variance, respectively. YR stripe rust, LR leaf rust, PM powdery mildew, FHB Fusarium head blight, GRY grain yield, KPS kernels per spike, TKW 1000 kernel weight, ESM ears per square meter, BM biomass, HI harvest index.

Results of correlation analyses between disease and yield traits at both N levels are summarized in Table 3 and Fig. 1. In general, factorial relation and correlation between parameters of diseases, grain yield and yield components were similar at both N levels. Factor PCA indicated that GRY, BM, HI and TKW formed a cluster at the first two principal components, which explain 39 to 44% (PC 1 at 110 and 220 kg N ha^−1^) and 23% or 22% (PC 2 at 110 and 220 kg N ha^−1^) of the phenotypic variance. Accordingly, GRY, KPS, BM, and HI correlate very strongly with each other at both N levels. In contrast, yield component ESM shows only a weak positive correlation to GRY, BM, and HI at both N levels but no correlation to KPS and a weak negative or no correlation to TKW. With regard to diseases significant positive correlations were observed between YR and FHB (110 kg N ha^−1^ r = 0.25, 220 kg N ha^−1^ r = 0.46) and between YR and PM (110 kg N ha^−1^ r = 0.42, 220 kg N ha^−1^ r = 0.22). Highly significant negative correlations were detected between YR and LR (110 kg N ha^−1^ r = − 0.40, 220 kg N ha^−1^ r = − 0.45) indicating strong competition between the two rust pathogens. Correlations between LR and the other diseases as well as between LR with GRY and the yield components were relatively weak or not significant, likely due to the abundance of YR. Pairwise significant negative correlation coefficients between all fungal diseases taken together (Fungal sum) and GRY at both N rates (110 kg N ha^−1^ r = − 0.67, 220 kg N ha^−1^ r = − 0.76) clearly demonstrate the impact of fungal diseases on yield. In other words, approx. 50% of the differences in grain yield can be explained by the additive or combined effect of the fungal pathogens. Likewise, the cluster of GRY and yield-related parameters had a factor load opposite to YR at both N levels and additionally opposite to PM at low N and FHB at high N (Fig. 1). In detail, highly significant negative correlations were observed primarily between GRY and YR (110 kg N ha^−1^ r = − 0.80, 220 kg N ha^−1^ r = − 0.86) and between GRY and FHB (110 kg N ha^−1^ r = − 0.42, 220 kg N ha^−1^ r = − 0.64). Correlations between GRY and PM were highly significant but lower, particularly at higher N rate (110 kg N ha^−1^ r = − 0.60, 220 kg N ha^−1^ r = − 0.36). Correlation between LR and GRY (r − 0.37) was obtained by partial correlation analysis taking into account those cultivars with low YR infestation (YR_1_ < 4.0455%, low or no competition between YR and LR). Partial correlation coefficients between LR and GRY are in general declining in classes with higher YR infestation and are not significant in class YR_5_ (> 13.242%) supporting the notion of a YR dominance effect in YR susceptible cultivars (cf. Tables 3, 4). Partial correlation coefficients between LR and GRY are in general declining in classes with higher YR infestation.

**Table 3:**
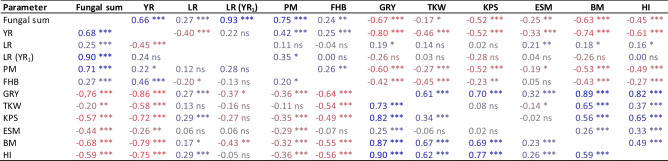
Correlation matrix for fungal diseases (YR, LR including subgroup of LR with low YR infestation, PM, FHB), yield (GRY) and yield components (TKW, KPS, ESM, BM, HI) based on LSmeans (observations over years and repeats) at two N rates, with 110 kg N ha^−1^ above diagonal and 220 kg N ha^−1^ below diagonal. Colours indicate degree and direction of correlation.

Fungal sum, YR + LR + PM + FHB; YR, stripe rust; LR, leaf rust; LR (YR_1_), leaf rust at quantile class with YR < 4.0455%; PM, powdery mildew; FHB, Fusarium head blight; GRY, grain yield; TKW, 1000 kernel weight; KPS, kernels per spike; ESM, stand density/m^2^; BM, aboveground biomass; HI, harvest index; Signif. p, significance level of p value: *** < 0.001, ** < 0.01, * < 0.05, ns not significant.

**Table 4:** Bivariate analysis between diseases (YR, LR including LR by YR classes, PM, FHB), and yield (GRY) based on LSmeans (observations treatment-specific, over years and repeats) assuming a linear model with variance explained (R^2^) and significance levels of p values of each model tested, and equations of linear fit to estimate grain yield from values of each disease.

Parameter treatment	R^2^	Equation to estimate GRY
**Fungal sum (YR + LR + PM + FHB)**		
T1	0.454***	GRY (110) = 7.80–0.1016*Fungal sum
T3	0.571***	GRY (220) = 7.928–0.123*Fungal sum
All	0.564***	GRY = 8.064–0.1222*Fungal sum
**YR**		
T1	0.460***	GRY (110) = 6.748–0.1346*YR
T3	0.530***	GRY (220) = 6.191–0.1527*YR
All	0.453***	GRY = 6.514–0.1497*YR
**LR**		
T1		Partial analysis, see below
T1 (YR_1_)	0.102*	GRY (110, YR_1_) = 6.732–0.04959*LR
T1 (YR_2_)	0.042 ns	GRY (110, YR_2_) = 6.366–0.04032*LR
T1 (YR_3_)	0.044 ns	GRY (110, YR_3_) = 6.091–0.05788*LR
T1 (YR_4_)	0.013 ns	GRY (110, YR_4_) = 5.094–0.03269*LR
T1 (YR_5_)	0.030 ns	GRY (110, YR_5_) = 5.006–0.1132*LR
T3		Partial analysis, see below
T3 (YR_1_)	0.138*	GRY (220, YR_1_) = 6.4444–0.06995*LR
T3 (YR_2_)	0.060 ns	GRY (220, YR_2_) = 5.956–0.05506*LR
T3 (YR_3_)	0.010 ns	GRY (220, YR_3_) = 5.095–0.02508*LR
T3 (YR_4_)	0.009 ns	GRY (220, YR_4_) = 4.639–0.02675*LR
T3 (YR_5_)	0.004 ns	GRY (220, YR_5_) = 3.566–0.03022*LR
All		Partial analysis, see below
All, (YR_1_)	0.153***	GRY (YR_1_) = 6.685–0.07277*LR
**PM**		
T1	0.255***	GRY (110) = 6.492–0.1716*PM
T3	0.247***	GRY (220) = 5.969–0.1867*PM
All	0.273***	GRY = 6.343–0.2026*PM
**FHB**		
T1	0.057**	GRY (110) = 6.214–0.2173*FHB
T3	0.116***	GRY (220) = 5.853–0.3048*FHB
All	0.155***	GRY = 6.275–0.3603*FHB

T1, T3, treatment 1, treatment 3; YR, stripe rust; LR, leaf rust; YR_1_-YR_5_, quantile classes of YR infestation (YR_1_ < 4.0455%, YR_2_ 4.0455 to 6.1509%, YR_3_ 6.1509 to 9.1852%, YR_4_ 9.1852 to 13.242%, YR_5_ 13.242 to 31%); PM, powdery mildew; FHB, Fusarium head blight; GRY, grain yield; Signif. p, significance level of p value: *** < 0.001, ** < 0.01, * < 0.05, ns not significant.

All leaf diseases, including LR at 220 kg N ha^−1^ and low YR abundance, were also negatively correlated with the yield component KPS, with BM, and HI and to a lesser extend with TKW. In comparison, FHB shows higher correlations mainly with GRY, TKW, and BM at low N, but additionally also with KPS at high N supply. ESM is slightly negatively correlated with YR (110 kg N ha^−1^ r = − 0.33, 220 kg N ha^−1^ r = − 0.26), significantly but weaker with PM (110 kg N ha^−1^ r = − 0.19, 220 kg N ha^−1^ r = − 0.29), and not with LR (class with lowest YR infestation) and FHB. PM correlates only moderately negatively with TKW at low N, but no correlation was observed at high N. That is in contrast to the other fungal diseases, which show negative correlations with TKW at both levels of N input.

**Figure 2:**
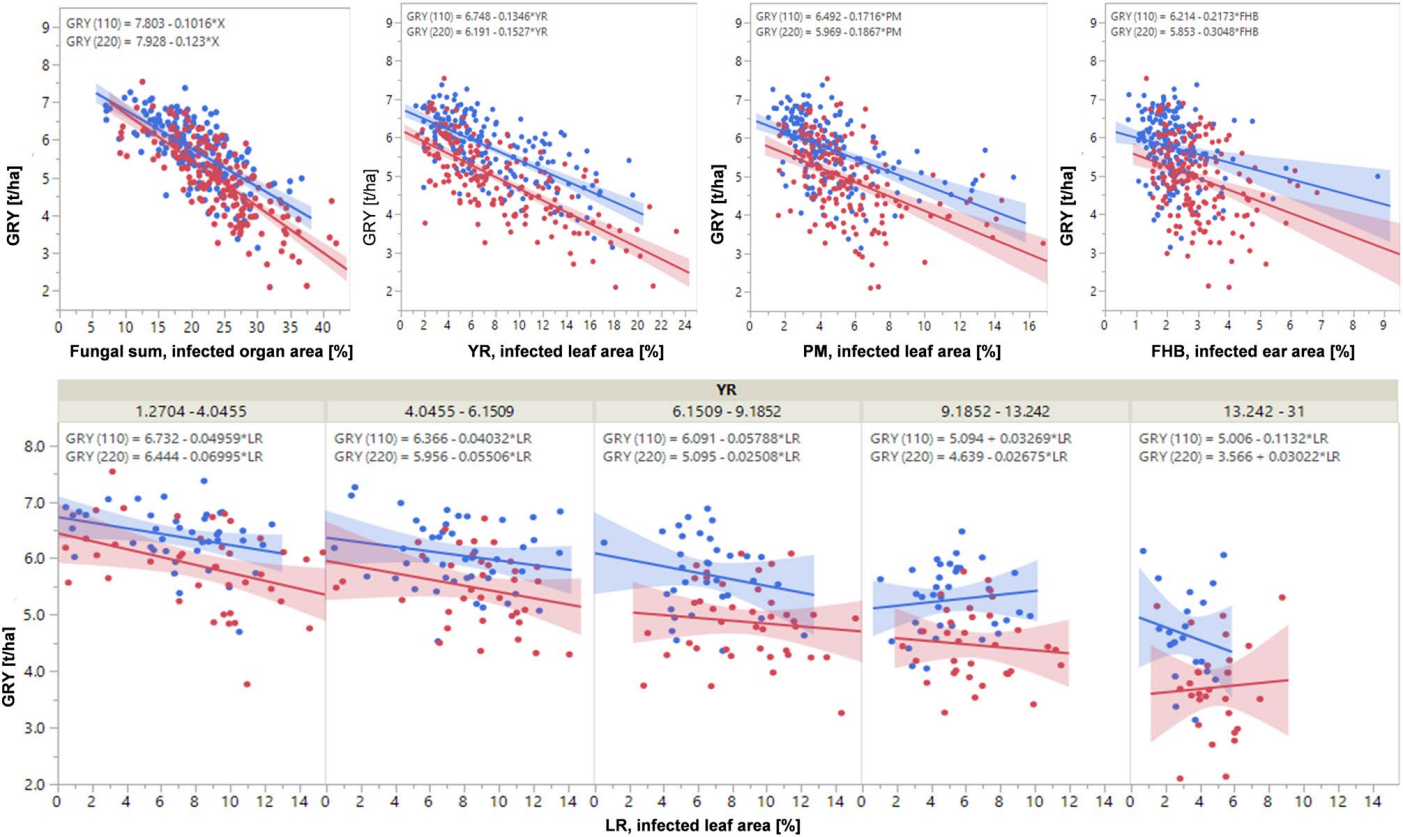
Bivariate analysis of grain yield (GRY, t/ha) in relation to fungal diseases as a group (Fungal sum), stripe rust (YR), powdery mildew (PM), and Fusarium head blight (FHB; all above), and partial bivariate analysis (below) of grain yield (GRY) in relation to leaf rust (LR) at five classes of YR infestation based on LSmeans (treatment-specific, over years and repeats). Each pair of analysed parameters with linear trend lines of low N (blue 110 kg N ha^−1^) and high N input (red 220 kg N ha^−1^), each trendline with 95% confidence interval. Equations of all trendlines are given above each plot.

Bivariate analysis indicates substantial yield losses resulting from fungal diseases (Table 4, Fig. 2). Using the slope of the regression line as an estimator, per basis point of visible pathogen symptoms (on leaves or spike) an average GRY loss of 102 kg ha^−1^ at 110 kg N ha^−1^ resp. 123 kg ha^−1^ at 220 kg N ha^−1^ is expected. More specifically, average GRY loss per % tissue damage caused by YR is 135 kg ha^−1^ (110 kg N ha^−1^) resp. 153 kg ha (220 kg N ha^−1^). LR (YR_1_), based only on cultivars with low YR infestation results in a loss of 50 kg ha^−1^ (110 kg N ha^−1^), resp. 70 kg ha^−1^ (220 kg N ha^−1^). PM (172 kg ha^−1^ at 110 kg N ha^−1^, 187 kg ha^−1^ at 220 kg N ha^−1^) and FHB (217 kg ha^−1^ at 110 kg N ha^−1^ and 305 kg ha^−1^ at 220 kg N ha^−1^) cause more severe yield losses than both rust diseases.

Data indicate a steady increase in yield gain due to fungicides (T4 minus T3; T2 minus T1) along with an increasing sum of disease scores (Fungal sum) at both N levels. Even the most resistant cultivars show a positive response to fungicides. If a linear dependency between yield gain to fungicides and the sum of disease scores is assumed (linear fit: T2-T1 = 0.642 + 0.0473*T1_fungal_sum; V4-V3 = 0.999 + 0.0712*V3_fungal_sum), a yield gain due to fungicides alone of + 0.64 t ha^−1^ at the low N rate and + 1.00 t/ha^−1^ can be inferred.

### Breeding progress for disease resistance and its relation to yield enhancement

**Figure 3:**
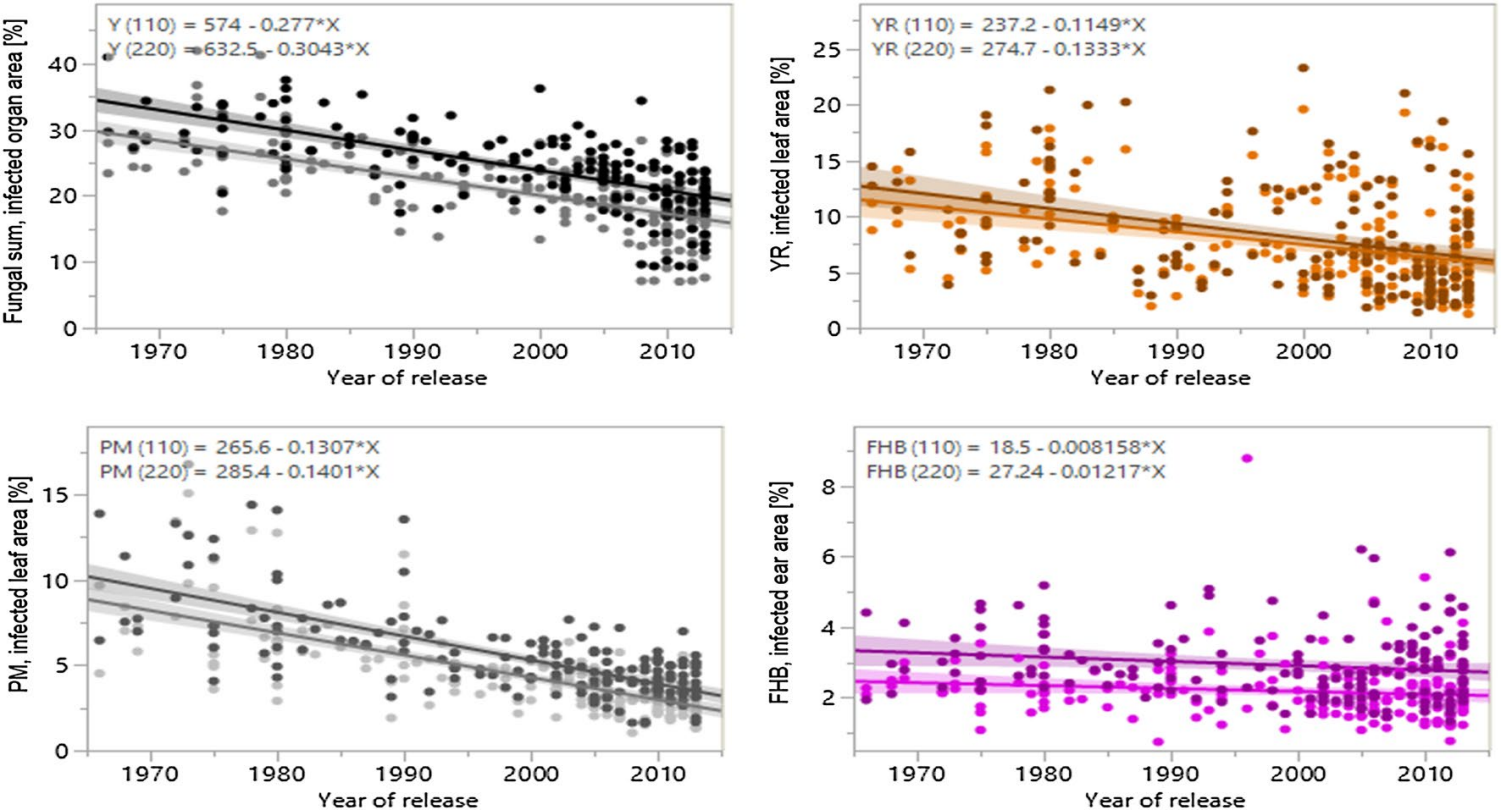
Breeding progress in German winter wheat against fungal pathogens. Development of susceptibility to four fungal pathogens (Fungal sum), stripe rust (YR), powdery mildew PM), and Fusarium head blight (FHB) of 178 winter wheat cultivars across 50 years at two N rates (treatments without plant protection) based on LSmeans (treatment-specific, over years and repeats). Linear trend lines of each disease is given for low N (110 kg N ha^−1^, lighter colour) and high N input (220 kg N ha^−1^, darker colour), each with 95% confidence interval. Equations of all trendlines are given above each plot.

**Figure 4:**
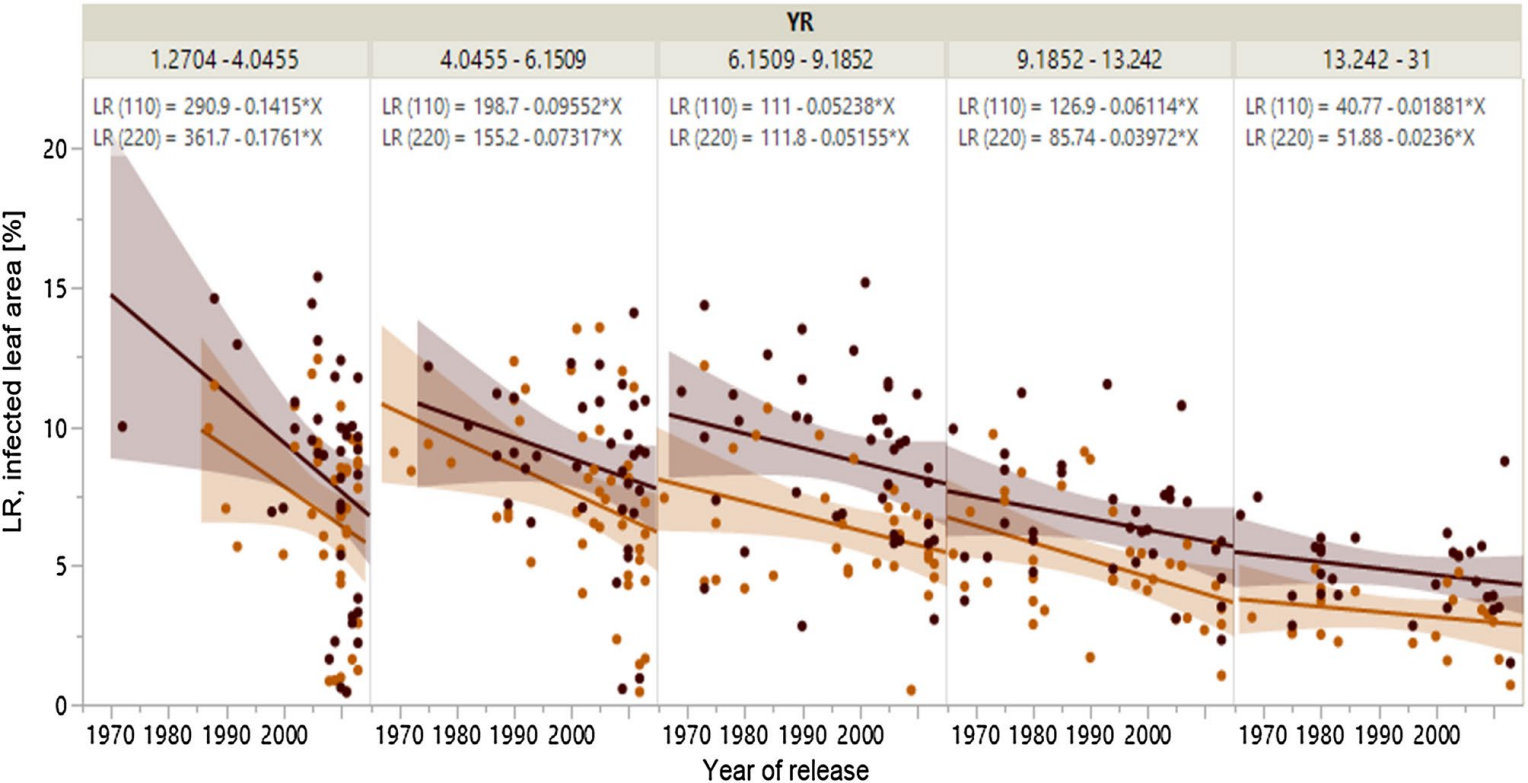
Breeding progress in German wheat against leaf rust. Development of susceptibility of 178 winter wheat cultivars to leaf rust (LR) depending from stripe rust (YR) infestation across 50 years at two N rates (treatments without plant protection) based on LSmeans (treatment-specific, over years and repeats). LR infestation of each YR class is given with linear trend lines of low N (110 kg N ha-1, light brown) and high N input (220 kg N ha^−1^, dark brown), each with 95% confidence interval. Equations of all trendlines are given above each plot.

**Figure 5:**
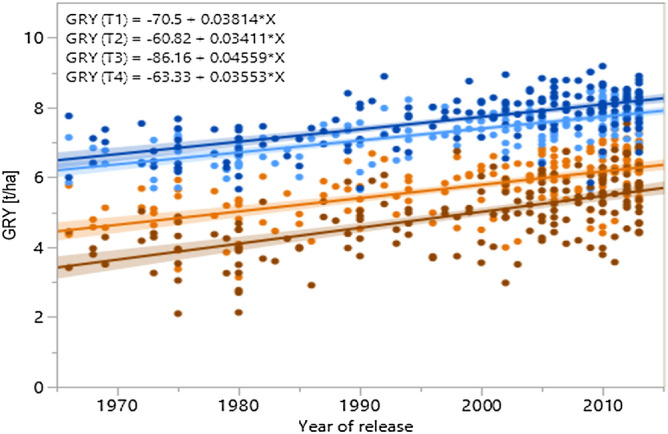
Breeding progress in German winter wheat. Progress of grain yield (GRY) of 178 winter wheat cultivars across 50 years at four treatments (T1 low N, no fungicide orange, T2 low N with fungicide light blue, T3 high N no fungicide brown, T4 high N with fungicide dark blue) based on LSmeans (treatment-specific, over years and repeats). Trendlines of GRY progress are given of all treatments, each with 95% confidence interval. Equations of all trendlines are given above each plot.

All four fungal diseases correlated significantly negatively with the year of cultivars’ release (Table 5, Figs. 3, 4, 5, c.f. Supplementary Table S4). The average rate of susceptibility over time, estimated from the slope of linear regression, decreased for all four fungal diseases, indicating sustained breeding progress. Moreover, the progress in general fungal resistance was slightly stronger at high N input (− 0.28% infestation per year at 110 kg N ha^−1^ vs. − 0.30% a^−1^ at 220 kg N ha^−1^). Specifically, susceptibility to PM (110 kg N ha^−1^: − 0.13% a^−1^; 220 kg N ha^−1^: − 0.14% a^−1^), and YR (110 kg N ha^−1^: − 0.11% a^−1^, 220 kg N ha^−1^: − 0.13% a^−1^) was notably and significantly reduced. Although a decreasing slope cannot be shown for LR based on a simple bivariate analysis, it can be inferred from the partial analysis of LR. In LR (YR_1_) the class of low YR infestation, if the competitive influence of YR is largely excluded, LR decreases considerably (110 kg N ha^−1^− 0.14% a^−1^ not significant; 220 kg N ha^−1^: − 0.18% a^−1^). Susceptibility against FHB decreased slightly (110 kg N ha^−1^: − 0.008% a^−1^, not significant; 220 kg N ha^−1^− 0.012% a^−1^).

**Table 5:** Bivariate analysis between diseases (YR, LR including LR by YR classes, PM, FHB), and yield (GRY) with years of cultivars release based on LSmeans (observations treatment-specific, over years and repeats) assuming a linear model. Results contain phenotypic variance explained (R^2^), significance levels of p values of each model tested, equations of linear fit to estimate breeding progress from year of release (YoR), and relative annual improvement of each parameter between 1965 and 2013 (estimated value of 1965 set to 100%).

Parameter treatment	R^2^	Equation to estimate parameter	Relative annual improvement 1965 to 2013 (%)
**Fungal sum (YR + LR + PM + FHB)**			
T1	0.451***	Fungal sum (110) = 574–0.277*YoR	− 0.932
T3	0.439***	Fungal sum (220) = 632.5–0.3043*YoR	− 0.881
All	0.401***	Fungal sum = 603.2–0.2906*YoR	− 0.903
**YR**			
T1	0.14***	YR (110) = 237.2–0.1149*YoR	− 1.005
T3	0.14***	YR (220) = 274.4–0.1333*YoR	− 1.069
All	0.138***	YR = 256–0.1241*YoR	− 1.022
**LR**			
T1		Partial analysis, see below	
T1 (YR_1_)	0.089 ns	LR (110, YR_1_) = 290.9–0.1415*YoR	− 1.101
T1 (YR_2_)	0.130**	LR (110, YR_2_) = 198.7–0.09552*YoR	− 0.868
T1 (YR_3_)	0.113 ns	LR (110, YR_3_) = 111–0.05238*YoR	− 0.648
T1 (YR_4_)	0.189**	LR (110, YR_4_) = 126.9–0.06114*YoR	− 0.904
T1 (YR_5_)	0.04 ns	LR (110, YR_5_) = 40.77–0.01881*YoR	− 0.495
T3		Partial analysis, see below	
T3 (YR_1_)	0.128*	LR (220, YR_1_) = 361.7–0.1761*YoR	− 1.124
T3 (YR_2_)	0.007 ns	LR (220, YR_2_) = 155.2–0.07317*YoR	− 0.641
T3 (YR_3_)	0.057 ns	LR (220, YR_3_) = 111.8–0.05155*YoR	− 0.490
T3 (YR_4_)	0.070 ns	LR (220, YR_4_) = 85.74–0.03972*YoR	− 0.516
T3 (YR_5_)	0.048 ns	LR (220, YR_5_) = 51.88–0.0236*YoR	− 0.430
All (YR_1_)	0.108**	LR (YR1) = 333.9–0.1626*YoR	− 1.130
**PM**			
T1	0.521***	PM (110) = 265.6–0.1307*YoR	− 1.490
T3	0.512***	PM (220) = 285.4–0.1401*YoR	− 1.387
All	0.494***	PM = 275.5–0.1354*YoR	− 1,435
**FHB**			
T1	0.022 ns	FHB (110) = 18.5–0.008158*YoR	− 0.331
T3	0.027*	FHB (220) = 27.24–0.01217*YoR	− 0.366
All	0.017**	FHB = 22.87–0.01016*YoR	− 0.350
**GRY**			
T1	0.38***	GRY (T1) = − 70.5 + 0.03814*YoR	+ 0.858
T2	0.47***	GRY (T2) = − 60.82 + 0.03411*YoR	+ 0.548
T3	0.38***	GRY (T3) = − 86.16 + 0.04559*YoR	+ 1.334
T4	0.42***	GRY (T4) = − 63.33 + 0.03553*YoR	+ 0.546
T1, T2 (low N)	0.19***	GRY (low N) = − 65.66 + 0.03613*YoR	+ 0.677
T1, T3 (without PP)	0.32***	GRY (wo PP) = − 78.33 + 0.04187*YoR	+ 1.061
T3, T4 (high N)	0.11***	GRY (high N) = − 74.74 + 0.04056*YoR	+ 0.818
T2, T4 (PP)	0.42***	GRY (PP) = − 62.07 + 0.03482*YoR	+ 0.549
All	0.138***	GRY = − 70.2 + 0.03834*YoR	+ 0.745

T1—T4, treatment 1 to 4; YR, stripe rust; LR, leaf rust; YR_1_-YR_5_, quantile classes of YR infestation (YR_1_ < 4.0455%, YR_2_ 4.0455 to 6.1509%, YR_3_ 6.1509 to 9.1852%, YR_4_ 9.1852 to 13.242%, YR_5_ 13.242 to 31%); PM, powdery mildew; FHB, Fusarium head blight; GRY, grain yield; Signif. p, significance level of p value: *** < 0.001, ** < 0.01, * < 0.05, ns not significant.

Breeding success in terms of healthier cultivars is also indicated by the best cultivars and their respective year of release (c.f. Supplementary Table S3 and S4). The healthiest cultivars against all fungal diseases (fungal sum) in the panel at both N levels were ‘SY Ferry’ (released 2012), ‘Xanthippe’ (2011), and ‘Zappa’ (2009). The cultivars with the lowest mean YR infestation at 110 kg N ha^−1^ were ‘Desamo’ (2013), ‘Nelson’ (2011) and ‘SY Ferry’ (2012) while ‘Zappa’ (2009), ‘Torrild’ (2005) and ‘Edgar’ (2010) were the best at 220 kg N ha^−1^. ‘Capone’ (2012), ‘Xanthippe’ (2011), and ‘Hyland’ (2009) at 110 kg N ha^−1^ and ‘Xanthippe’ (2011), ‘Hyland’ (2009), and ‘Muskat’ (2010) at 220 kg N ha-1 showed the lowest LR infection. With regard to PM ‘Tabasco’ (2008), ‘Memory’ (2013), and ‘Anapolis’ (2013) at 110 kg N ha^−1^ and ‘Primus’ (2009), ‘Tabasco’ (2008), and ‘Edward’ (2013) at 220 kg N ha^−1^ were the best cultivars, nearly without infestation. ‘Greif’ (1989), ‘Elixer’ (2012), and ‘Chevalier’ (2005) at 110 kg N ha^−1^ and ‘Elixer’ (2012), ‘Esket’ (2007) and ‘Dekan’ (1999) were the cultivars least affected by FHB.

Although the average progress for overall resistance against all fungal pathogens tested was higher at 220 kg N ha^−1^ than at 110 kg N ha^−1^ in absolute terms, it was very similar at different N levels on a relative basis (− 0.93% a^−1^ at 110 kg N ha^−1^ and− 0.88% a^−1^ at 220 kg N ha^−1^; starting 1965 set to 100%, Table 5). Relative breeding progress against PM (110 kg N ha^−1^: − 1.49% a^−1^; 220 kg N ha^−1^: − 1.39% a^−1^) was very strong. Considerable improvement can also be found for resistance against YR (110 kg N ha^−1^: − 1.00% a^−1^, 220 kg N ha^−1^: − 1.07% a^−1^) as well as against LR (YR_1_) (110 kg N ha^−1^: − 1.10% a^−1^ und 220 kg N ha^−1^: − 1.12% a^−1^; both in cultivars with low YR), but was relatively weak against FHB (110 kg N ha^−1^: − 0.33% a^−1^; 220 kg N ha^−1^: − 0.37% a^−1^).

Breeding gain for GRY was highly significant in all production systems (treatments) with an average rate of + 38.3 kg ha^−1^ a^−1^ (T1 + 38.1 kg ha^−1^ a^−1^, T2 + 34.1 kg ha^−1^ a^−1^, T3 + 45.6 kg ha^−1^ a^−1^, T4 + 35.5 kg ha^−1^ a^−1^; Table 4, Fig. 5). GRY increase was more pronounced in treatments without plant protection (T1, T3) and strongest without plant protection at high N fertilization (T3, Fig. 5). Annual gain in GRY over the last 50 years on a relative basis was + 0.75% a^−1^, ranging from moderate + 0.55% a^−1^ at both treatments with plant protection independent from N input (T2, T4) to a steeper increase (+ 0.86% a^−1^) at the low intensity treatment (T1) and the highest increase (+ 1.33% a^−1^) without plant protection at high N fertilization (T3). Mostly recent cultivars, such as ‘Tobak’ (2011), ‘Memory’ (2013), and ‘Elixer’ (2012) gave the best yield at treatment T1, combining high yield potential at low N and low yield damage by fungal pathogens. ‘Hyland’ (2009), ‘Premio’ (2006), and ‘Potenzial’ (2006) yielded best at the protected low N treatment T2. ‘Elixer’ (2012), ‘Gordian’ (2013), and ‘Desamo’ (2013) produced the highest yield without protection at high N (T3), and ‘Hyland’ (2009), ‘Kalahari’ (2010), and ‘Winnetou’ (2002) at T4 with fungicides, respectively. The best yield performance to all treatments, exhibiting the highest flexibility to the production system, gave ‘Gordian’ (2013), ‘Elixer’ (2012), and ‘Patras’ (2012).

## Discussion

It is well known that the rate of nitrogen supply can alter the susceptibility of crop plants to fungal pathogens, but the effects depend on the host, the type of pathogen (biotrophic vs. necrotrophic)^59,60^, and the time of N application^61^. As the impact of N supply on crop diseases is complex, it was argued that it should be investigated in a crop- and pathogen-specific manner^62^.

In the present study four major fungal pathogens, varying in their phenological niche and severity, were used to assess a panel of historically and agronomically important cultivars of German winter wheat at two levels of N supply, resembling relevant low and high input conditions. Resistances and yield traits were analyzed at each N level and in control treatments in which diseases were excluded by extensive fungicide spraying. Substantial variation was detected for all resistances and yield traits. It confirms previous findings that N fertilization tends to increase the number of ears per m^2^, total biomass, kernels per spike, and grain yield, but reduces TKW if fungal diseases are absent^58,63^. As ears per m^2^ and biomass are elevated at higher N supply, it can be concluded that also the microclimate was more favorable to fungal pathogens. Accordingly, infestations by all four fungal pathogens analyzed were responsive to the N level (Table 1). Our results support the previous finding that biotrophic fungal pathogens (i.e. *Blumeria graminis, Puccinia striiformis*, *P. triticina*)) benefit from increased N supply^13,64,65^. The hemibiotrophic pathogen *Fusarium culmorum* (with a biotrophic and a necrotrophic phase) increases strongly with higher N as well, supporting the findings of^40^ who reported increasing FHB severity with higher N fertilization rates from 0 to 160 kg N ha^−1^ (independent from the type of fertilizer). Although we found a broad genotypic variation among the cultivars ranging from nearly resistant to highly susceptible to all pathogens tested, G × N interaction was not detected for stripe rust (YR), leaf rust (LR), and powdery mildew (PM) infestation. That suggests that the cultivars analyzed respond similarly to increased N fertilization.

The abundance of YR was relatively high, which can be attributed to the fact that a mixture of relatively new and aggressive *P. striiformis* races such as ‘Warrior’, ‘Warrior (−)’, ‘Triticale aggressive’ and ‘Oakley, v7/Kranich’ have been used for inoculation. Most resistance genes deployed in the European breeding genepool are overcome by these highly virulent races, including *Yr6* by all four races, *Yr8* and *Yr10* by ‘Triticale aggressive’, *Yr1*, *Yr2*, *Yr3*, *Yr7*, *Yr9*, *Yr17*, *Yr25* and *Yr32* by ‘Oakley, v7Kranich’ and the Warrior races, and *YrSd* as well as *YrSP* by the Warrior races^21,66,67^. There was at least some YR infestation in even the most resistant cultivars, e.g. ‘Desamo’, ‘Nelson’ or ‘SY Ferry’ (Supplementay Table S3). As qualitative resistance should in general not result in infestation quantitative resistance most probably caused YR differences detected on the panel. This observation is in accordance with Hovmøller^66^ who assumed that the qualitative resistance genes *Yr5*, *Yr24*, and *Yr15,* which are still functional, are very likely not deployed in German elite wheat varieties.

Some genes are known to confer to quantitative resistance against YR and LR, namely *Lr34*, *Lr46*, *Lr67*, *Lr68*, and likely also *Yr17*. It is not known if German cultivars contain the less common quantitative resistance genes *Lr67* and *Lr68* as breeders often hide such information and differential sets to study *Lr* genes usually do not cover these genes up to now (c.f. Zetzsche et al.^21^). Most common leaf rust isolates are able to overcome *Lr46*. However, *Lr46* might have contributed to increased quantitative resistance in modern cultivars present in our panel via combination of several resistance loci/genes^21^. No dedicated study on the occurrence of the more common slow rusting *Lr34*/*Yr18* region exists for German cultivars. However, ‘Kormoran’ (part of our study, released 1973 by Lochow-Petkus, predecessor of KWS) containing *Yr18* and *Yr17* is relatively common in German cultivars according to the Wheatpedigree database (www.wheatpedigree.net). It seems likely that *Yr17* and/or *Lr34*/*Yr18* together with further quantitative resistance loci/genes have been combined in the German elite cultivars as KWS released many successful cultivars with good overall resistance after the year 2000 and other breeders are allowed taking advantage from resistant cultivars through the breeder’s privilege. Leaf tip necrosis (LTN) has been described to be associated with some of these quantitative resistance genes. LTN occurred at varying degree in most of the cultivars in 2015. It significantly increased in both protected treatments, T2 and T4, over the five decades along with the increase in disease resistance. Thus, increased LTN could result from increased disease resistance due to a combination of quantitative resistance genes.

Higher N fertilization increases the susceptibility of wheat against YR supporting previous studies^13,14^, but G × N interaction was not shown to be significant. In other words, the cultivars react very similar to higher N supply. Nevertheless, with respect to breeding progress varietal YR susceptibility was reduced considerably in the course of decades from an average of 11.4% leaf area infected to 5.9% at 110 kg N ha^−1^ and from 12.5% to 6.1% at 220 kg N ha^−1^ (cf. Fig. 3). Although the absolute reduction rate was steeper at the higher N level, the rate of relative improvement (starting in 1965 with the infected leaf areas each set to 100%) equals to a constant rate of 1.0% per year at both N levels. The significant decrease of infestation by YR despite continuous breakdown of previously introduced resistance genes during the observed time interval supports the hypothesis that at least some of the R genes, which mediate qualitative resistance, may contribute also to quantitative resistance already^21,68^. Additionally, accumulation of durable quantitative YR resistance loci may have contributed to improved resistance in the field^69^.

In comparison to YR a lower average leaf rust (LR) susceptibility was observed, but the yearly abundance of both rusts differed considerably (cf. Table 1). The increase of mean LR infestation due to higher N fertilization was pronounced which is in agreement with results of previous studies^22–24^. Being inoculated onto the same wheat plants LR mainly developed on cultivars with a low susceptibility to YR. As a result, LR and YR disease expressions showed a strong negative correlation. It may be hypothesized that LR infestation is competitively suppressed by YR as both foliar fungal pathogens rival for a very similar ecological niche where YR has the advantage of first occupation. Competition is supported by the fact that both diseases do not correlate with each other if YR abundance is low, and partial correlation coefficients decrease along with an increase of YR infestation.

Older cultivars in the panel were more often infested by YR and because of the negative correlation to LR do not allow an unbiased estimate of LR infestation. Hence, partial correlation analysis of cultivar classes with similar YR susceptibility (Fig. 4) shows that LR resistance has been strongly improved over the past five decades as well. Relative improvement of LR resistance at a rate of 1.1% per year is independent from N input and only slightly higher than the improvement against YR. Ahlemeyer and Friedt^51^ found a lower relative annual raise of resistance at 0.6% for LR between 1966 and 2007 based on data from official trials by the German Plant Variety Office analyzed over that period. The difference might be attributable to the different trial types. Our rates of improvement are based on joint trials which subsume durable resistance progress and resistance which has been lost by breakdown of resistance genes^54^. The calculations of Ahlemeyer and Friedt^51^ were based on data of continuing official German variety tests for cultivation and use (VCU^70^) and reflect durable resistance progress only. If durable progress is assumed to be equal for both types of trials, subtracting the rate of durable resistance progress from the combined rate gives a rough annual estimate for the relative annual breakdown of resistance genes of 0.5% for LR.

Susceptibility to powdery mildew (PM) was also considerably increased by higher N fertilization. As plant density and biomass was increased due to higher N, it strongly supports previous reports according to which dense sowing and high N supply can increase susceptibility to PM^23,71–74^. Although no artificial inoculation with PM was carried out, natural infection occurred regularly with at least low abundancy in late fall of each year. The disease severely progressed under humid and warm weather conditions (e.g. in May and July 2017) when even the least susceptible cultivars such as ‘Tabasco’, ‘Zappa’ or ‘Memory’ showed diseases symptoms. PM remains a constant threat to wheat despite the fact that resistance over the past five decades was improved strongly with a decrease of susceptibility from 8.8% to 2.5% leaf area infected at 110 kg N ha^−1^ and from 10.1% to 3.4% at 220 kg N ha^−1^ (cf. Fig. 3). That equals to a relative improvement of 1.49% a^−1^ and 1.39% a^−1^, respectively. The relative rate of improvement in resistance according to Ahlemeyer and Friedt^51^ was 1.46% a^−1^, almost identical with the rate which we observed. Considering that the resistance progress levelled off in the mid 2000′s, recalculation gives a rough estimate of 0.7% (110 kg N ha^−1^) resp. 0.5% (220 kg N ha^−1^) for PM to compensate for the average annual rate of breakdown of resistance, similar to the value for LR. Our results support previous studies, that intense wheat resistance breeding achieved sustained resistance^4,52, 58^.

The N application rate had a significant impact on the susceptibility to Fusarium head blight (FHB), as infestation at 220 kg N ha^−1^ was considerably higher than at the lower N supply. FHB symptoms developed rapidly after artificial inoculation with the highly virulent isolate Fc46^75^ adjusted to the flowering time of cultivars and followed by two subsequent water sprayings. We found significant G × N interaction for FHB suggesting that cultivars react differently to the pathogen at different N levels. This is in contrast to previous studies, in which no significant effects of varying N rates on FHB susceptibility^43,44^ or even contradicting data^42^ were reported. As expected, only quantitative differences of susceptibility were detected. In contrast to YR, PM, and LR, were modern cultivars mostly belong to the least susceptible, breeding progress for FHB resistance is not obvious by years of cultivar’s release of the best in comparison to the most susceptible cultivars. However, average infestation decreased slightly over time from 2.5% to 2.1% at 110 kg N ha^−1^ and from 3.3% to 2.7% at 220 kg N ha^−1^ (Fig. 3). If a linear trend is assumed, this results in a relative annual decrease in susceptibility of 0.33% a^−1^ at 110 kg N ha^−1^ and 0.37% a^−1^ 220 kg N ha^−1^. FHB resistance of European cultivars is predominantly quantitative, i.e. mediated by many loci with small effects^38,76^. It is generally argued that quantitative resistance is more durable and a (gradual) breakdown of such resistances is unlikely. If the latter would be assumed for the recalculation of the relative annual improvement, durable breeding progress for FHB resistance would be rather low.

The intensity of fungal diseases as a whole correlates significantly negatively with yield and single yield components (Table 3). The negative effect of fungal pathogens on the different yield components corresponds to the seasonal occurrence of the diseases. Early occurring diseases such as PM and YR significantly decrease crop density (number of ears per square meter) while late occurring diseases usually have no pronounced or significant effect. The number of kernels per spike is the yield component most strongly affected by the foliar diseases. This can be explained by the impairment of photosynthesis and reduction of assimilates impacting flower development, fertilization, and the development of ovules and intact seeds. In contrast, toxins related to the ear disease FHB mainly affect later stages of ovule development and seed formation, potentially leading to the abortion of developing ovules. FHB, YR, and LR have been very abundant mainly during grain filling, which explains their negative correlation with TKW. On the contrary, PM usually occurs earlier but also more permanently and seems to reduce grain yield primarily via the reduction of kernels per spike, biomass and harvest index.

A high N supply tends to promote ears per square meter and all four fungal diseases. In turn, correlations between ears per square meter and each of these diseases are generally low and difficult to interpret. Higher N supply leads to stronger negative correlation between YR, LR and grain yield, as well as for FHB with TKW and grain yield in comparison to a lower N supply. This supports the assumption of the source-based cause of a higher N supply leading to stronger disease infestation. Stronger infestation by fungal pathogens uses the plants’ assimilates and negatively affects photosynthesis, and organ development, which in turn damages yield components resulting in lower biomass formation and finally a reduction in grain yield. The expected loss of grain yield differs for each of the diseases but is in general increased at higher N supply (cf. Fig. 2). In the present study, the biotrophic rust diseases have caused yield losses of 50 up to 153 kg ha^−1^ per 1% tissue damage with lower losses by leaf rust occurring late in the season. Yield losses resulting from powdery mildew infection were higher (172–187 kg ha^−1^ per 1% tissue damage) likely due to a longer duration of PM infestation in comparison to the rusts. FHB has not only caused the most severe losses of GRY (217–305 kg ha^−1^ per % of damaged tissue) via a reduction in TKW, it also releases toxins such as Deoxynivalenol (DON) und Zearalenon (ZEA) which can cause a reduction of kernels per spike, particularly at an increased N level. As it might also lead to a total loss of a wheat harvest by toxic contamination, resistance against this disease should be of high priority in further wheat breeding.

As demonstrated, grain yield could be enhanced over the whole time period of five decades in all four production systems (Fig. 5) with an average of + 38.3 kg ha^−1^ a^−1^. Increasing grain yield over time without chemical plant protection (T1, T3) results both from enhanced genetic grain yield potential (T2 + 34.1 kg ha^−1^ a^−1^, T4 + 35.5 kg ha^−1^ a^−1^) as well as from positive and stabilizing effects of enhanced fungal resistance (Fig. 5). Based on the estimated slopes, improved fungal resistance accounts for 11% (+ 3.8 kg ha^−1^ a^−1^) at low N input and 28.5% (+ 10.1 kg ha^−1^ a^−1^) of the grain yield increase under intense N conditions. Thus, the strongest improvement of German wheat cultivars has been achieved by enhancing both, yield potential and fungal resistance at high input conditions without plant protection (T3). We found evidence for a positive yield response to fungicides at both N rates even in largely resistant cultivars. More precisely, susceptible varieties profit more from fungicides than resistant cultivars but even the most resistant cultivars show a positive response. Modern fungicides (without strobilurins which were not used) had a very positive effect on yield. Reasons might be protective effects of fungicides against not yet visible damage by fungal diseases (e.g. by hemibiotrophic pathogens) and an increase in wheat stress tolerance (reduction of ethylene and oxidative stress) which were described by Wu & Tiedemann^77^ and Zhang et al.^78^.

In our study we used an approach in which all cultivars were analyzed simultaneously in the same field trials; rather than the analysis of historical data from continued official variety trials (VCU trials). Genetic gains in trials of the former design tend to be overestimated in the variants without fungicide applications^79^, attributed to “variety ageing” which results from gradual resistance breakdown^79–81^. Laidig et al.^54^ calculated the genetic gain of winter wheat in German official variety trials for the period from 1983 to 2012 at + 1.15% a^−1^ in intensity 1 (without fungicide) and + 0.66% a^−1^ for intensity 2 (with fungicide). The difference between the two intensities is interpreted by a non-genetic, agronomic trend plus an effect called ‘variety ageing’ of 0.36% a^−1^. Earlier, in a similar study including cultivars released from 1966 to 2007 Ahlemeyer and Friedt^51^ found a similar trend albeit lower values of 0.63% a^−1^ (intensity 1) and 0.46% a^−1^ (intensity 2), respectively. The respective trends observed in our trials range between a higher relative grain yield gain of + 1.32% a^−1^ in variant T3 (comparable with intensity 1 in VCU trials) and a similar 0.54% a^−1^ in T4 (comparable with intensity 2). ‘Ageing’ as calculated by Laidig et al.^54^ assumes the predominant release of mostly resistant varieties which is unlikely as Ahlemeyer and Friedt^51^ have shown. Thus, we have to consider ‘ageing’ effects by gradual breakdown of resistances and durable resistance gains both taken together explaining the difference between treatments with and without fungicides. Ahlemeyer and Friedt^51^ used susceptibility data from historical field trials which reflect durable resistance gains whereas resistance gains in our trial represent both durable resistance gain plus the loss of resistance by breakdown of R genes (the latter equaling the ageing effect). Thus, the relatively strong difference in grain yield gain between T3 and T4 (and partly also between T2 and T1) may result from the combined effect of these two components of resistance improvement. Drought stress and severe biotic stress through fungal pathogen pressure by artificial inoculation may have contributed additionally to yield differences between treatments. We conclude that the genetic trend in our trial might be somewhat overestimated due to stress, comparable to a potential overestimation of the genetic trend at low input conditions as observed by previous authors^79,82,83^.

Genetic gains and ‘variety ageing’ (breakdown of resistance) can be estimated if both variants with and without fungicide treatment are carried out in one trial^81^. The effect of fungicide application then directly affects the outcome of comparative field trials^54^. If chemical plant protection is very strict and successful, genetic gains can be estimated reasonably. That was likely the case regarding YR, LR, and FHB in our trial. If by comparison the fungicide control is not fully effective, older cultivars are burdened stronger by diseases and genetic gains may be biased and over-estimated. Accordingly, average annual breakdown of resistance genes would be underrated which could to some extent be the case for PM in our experiments.

In summary, it can be stated that different fungal pathogens attack wheat at different time points causing varying degrees of crop damage and yield loss. Results allowed quantifying the impact of N fertilization on the infestation by the fungal pathogens and their effects on grain yield as well as yield components. Resistance against fungal pathogens has been improved significantly but at varying speed during the last decades. The slope of improvement of genetic resistance was higher against biotrophic pathogens than to the hemibiotrophic pathogen. Our analyses support the assumption of the accumulation of longer-lasting quantitative resistance loci along with the deployment of race-specific R genes against biotrophic pathogens. Improvement in resistance over time was steeper at the higher N level in absolute but very similar in relative terms. The results strongly indicate that a substantial increase of genetic yield potential has been achieved in addition to simultaneous efforts to improve resistance against different fungal pathogens. As a matter of fact, the trend to higher resistance against the different wheat pathogens does not show signs of slowing down. Therefore, current German elite winter wheat cultivars provide an excellent basis for further yield gain by cross-breeding ("combination breeding"). That will enhance a more environment-friendly, sustainable crop production in the future leading to wheat cultivars which are better suited for farmers’ needs and consumers’ preferences. Drought periods can reduce the effectiveness of N application. As weather extremes are predicted to increase due to climate change and drought periods might become a greater problem particularly in semiarid areas agronomic strategies including N application and seed rates surely need to be adapted accordingly.

## Material and methods

### Plant material

A total of 178 German winter wheat cultivars, released in Germany between 1965 and 2013 (Bundessortenamt, BSA) were selected for the study based on their superior economic and agronomic importance in wheat production. The panel therefore represents long-term wheat breeding activities in Germany. General cultivar information including breeders and year of official release are provided in Supplementary Table S1. The panel of cultivars subsumes the German cultivars investigated by Voss-Fels et al.^58^.

### Experimental site and trial design

The complete panel has been grown on the experimental field of the Julius Kuehn Institute in Quedlinburg, Germany (51.7694 N, 11.147 E, 140 m altitude). The soil type is a Chernozem with a silty loam texture (Loe 1a), an average humus content of 2.1% and a pH value of 7.1. The test site is characterized by subcontinental temperate climatic conditions influenced by its geographical range in the rain shadow of the Harz Mountains with a long-term average air temperature of 8.9 °C and 497 mm yearly precipitation, respectively. The vegetation periods of the three consecutive growing seasons (2014/15, 2015/16, 2016/17) were characterized by above average temperatures (+ 1.3 °C), an average annual precipitation deficit of 29 mm, with drought periods during spring and early summer in 2015 and 2017, and a relatively dry winter in 2016/17. Relative soil moisture was measured in 40 cm depth using PlantCare Mini-Logger XL sensors (PlantCare AG, Russikon, Switzerland) from April to harvest. Relative soil moisture was on average 29.8% (2015 29.3%, 2016 31.1%, 2017 29.1%) which indicates below-average water availability.

Field trials were conducted in yield plots (area 4.5 m^2^) in three consecutive growing seasons from 2014–2015 to 2017–2018. In each of the three year × location environments all 178 cultivars were sown side by side in a full factorial combination of high or low nitrogen levels combined with the presence or absence of fungicides, respectively, each in two replicates. To ensure comparable pathogen pressure, artificial inoculation has been carried out in each year. In total, 4272 plots were analyzed. The wheat cultivars were grown in a randomized incomplete block design with four treatments (T1: 110 kg N ha^−1^, no fungicide; T2: 110 kg N ha^−1^ + fungicide; T3: 220 kg N ha^−1^, no fungicide; T4: 220 kg N ha^−1^ + fungicide) in two replications, each. All trials were sown at a density of 330 seeds m^−2^ and received an herbicide (Fenikan^®^, Bayer AG, Germany) treatment shortly after emergence. All variants were fertilized by Calcium ammonium nitrate (CAN, adjusted for soil N_min_) up to three times from March to May at growth stages BBCH23–25, BBCH24-31, BBCH33-45 (BBCH scale^84^) until the intended target amount was reached. N application in 2015 and 2017 was time-delayed due to drought periods and, by extension, likely only partly effective. Plant height was controlled in all treatments by two applications of growth regulator (CCC 720^®^, Bayer AG, Germany; Medax Top^®^, BASF AG, Germany) in April and May. Fungal pathogens have been successfully controlled (infestation ≤ 1%) in treatments 2 and 4 by three annual fungicide applications (Capalo^®^, Adexar^®^, both BASF AG, Germany; Prosaro^®^, Bayer AG, Germany) between April and June. Fungicides, insecticides (Karate^®^ Zeon, Syngenta AG, Switzerland) and growth regulators have been applied due to standard recommendations in Germany. Specific management information of each year and trial including date and amount of application is given in Supplementary Table S2.

Fungicides (plant protection, PP) effectively prevented the development of fungal diseases within the non-inoculated treatments (T2, T4). Symptoms were not scored plotwise but monitored to apply fungicides timely. Accordingly, FHB was never observed in the fungicide treatments. The diseased leaf area did not exceed 1% for YR and LR in all three years, and for PM in 2015 and 2016. Only PM occurred up to 4% on single susceptible cultivars in T2 and T4 during summer 2017.

### Pathogen multiplication and inoculation

Pathogen multiplication of *P. striiformis* (PS, isolates ‘Warrior’, ‘Warrior (−)’, ‘Oakley, v7/Kranich’, ‘Triticale aggressive’ (cf. Zetzsche et al.^21^) was carried out at the JKI Braunschweig according to Bayles et al.^85^. *P. triticina* (PT, isolates: ‘77WxR’, ‘Tommi1’, ‘167/76wxr’, ‘4083’) were multiplied according to Serfling et al.^86^, and *F. culmorum* (FC, isolate: Fc46) at the JKI Quedlinburg according to Kopahnke et al.^87^.

Treatments T1 and T3 were artificially inoculated with mixtures of PS, and PT races (see above) once, and with FC two times, each at periods of favorable growing conditions of the specific pathogen (PS: mid-April, BBCH23–30 at 5 to 10 °C; PT: mid-May, BBCH33–40 at 15 to 25 °C; FC: flowering, BBCH63–69; each at humidity > 80%). For the rusts, all respective plots were inoculated with an oil-spore mixture using a hand-held spinning disc sprayer (ULVA +, Micron Sprayers, Herefordshire, UK). Suspensions with a ratio of 0.2 g rust spores per liter oil (Isopar M, ExxonMobil Chemical Company, Spring, TX, USA) were prepared, sufficient for 400 m^2^ plot area per liter. FC was inoculated by an aqueous suspension adjusted to about 300,000 conidia ml^−1^ (25 m^2^ plot area per liter). High abundance of BGT led to spontaneous natural infection.

### Disease phenotyping and morphological and agronomical traits

Susceptibility of the cultivars to each disease was scored regularly four times (FHB two times) during each growing season starting when the first symptoms were visible. Percentage of leaf area infected was estimated as described by Moll et al.^88^. FHB infection percentage was quantified as a combination of disease severity and disease incidence, referred to as FHB index. The area under the disease progress curve (AUDPC) was calculated for each cultivar and disease. AUDPC values were then used to compute the average ordinate of infestation (AO^89^) for each of the diseases (YR, LR, PM, FHB),

$${\text{AO }} = \, \left( {\sum^{{{\text{Ni}} - {1}}}_{{{\text{i}} = {1}}} \left( {{\text{y}}_{{\text{i}}} + {\text{y}}_{{{\text{i}} + {1}}} } \right)/{2 }* \, \left( {{\text{t}}_{{{\text{i}} + {1}}} - {\text{t}}_{{\text{i}}} } \right)} \right)/{\text{t}}_{{\text{p}}}$$

where (N) is the number of observations, the disease level at the ith observation is coded by (y_i_), time at the ith observation by (t_i_), and the total monitoring period in days is coded by (t_p_). Fungal sum was calculated by unweighted addition of AO values of all four diseases.

The number of ears per square meter (ESM) was calculated based on counting the ears in one running meter of one of the inner rows in each plot. A subsample consisting of all plants of a row length of 0.5 m from inside the plot was cut prior to harvest to determine the harvest index (HI). Each sample was oven-dried starting at 65° increasing to 105 °C overnight. HI was calculated plotwise as the ratio (%) of the grain to the aboveground biomass. Number of kernels per spike (KPS) was computed based on grain yield divided by the thousand kernel weight and number of spikes. Total grain yield (GRY) was measured by harvesting entire plots of each cultivar and replicate. Grain moisture was determined and grain yield was corrected to a standard moisture of 14%. About 500 random seeds were selected from all samples to determine thousand kernel weight (TKW) using a MARVIN Seed Analyzer (GTA Sensorik GmbH, Neubrandenburg, Germany). Aboveground biomass (BM) of plots was calculated based on GRY and HI.

### Statistical analysis

Descriptive and summary statistics, contrast statistic as well as analysis of variance (ANOVA) for each parameter were calculated using JMP 14.0.0^90^. The LSmeans contrast function as implemented in JMP 14.0.0 has been used to test orthogonal contrasts between treatments by means of F statistics in order to examine the effects of the fixed two N rates and two plant protection levels. ANOVA was computed applying a factorial design model including four treatments (two levels of N input times two levels of plant protection) within three years, and 178 cultivars using the PROC MIXED procedure of SAS 9.4^91^ as implemented in JMP 14.0.0. N input and plant protection levels were considered as fixed factors, cultivars (genotypes) and years as random. Treatment-specific adjusted means (LSmeans) of each cultivar were calculated prior to analyses of relationships among traits and between traits and year of cultivar release. PROC RANK via JMP was then used to build quantile groups (classes) of similar YR infestation (YR_1_ < 4.0455%, YR_2_ 4.0455 to 6.1509%, YR_3_ 6.1509 to 9.1852%, YR_4_ 9.1852 to 13.242%, YR_5_ 13.242 to 31%). Pearson’s correlation coefficients were calculated using the PROC CORR procedure of JMP 14.0.0/SAS 9.4^90,91^. Factorial analysis was carried out by means of principal component analysis (PCA) using the PROC FACTOR algorithm. Data were analyzed by means of bivariate analysis also using SAS 9.4 procedure in JMP 14.0.0^90,91^ to explore the relationships between GRY and the fungal diseases based on treatment-specific adjusted means as input values. To quantify the breeding progress year of cultivar release, considered as continuous variable, and treatment-specific adjusted means have been used. Partial bivariate analysis with YR classes (quantile groups) was used to dissect the relation of both GRY to LR and LR to year of cultivar release due to the LR dependency of YR. Separate analyses of each class was conducted.

## Supplementary information

Supplementary material 1

Supplementary material 2

Supplementary material 3

Supplementary material 4

## Data Availability

The dataset [QLB_BRIWECS_WW_fieldtrial_adjustMeans_treatments.csv] used for this study is available via the online data repository Zenodo [https://zenodo.org/deposit/3697514] with the digital object identifier https://doi.org/10.5281/zenodo.3697514. All other data used in the analyses are given in the Supplementary Information. Seed aliquots from the cultivars analyzed in the study are available from the corresponding author upon reasonable request for research purposes only.
